# Aerosol-based functional nanocomposite coating process for large surface areas

**DOI:** 10.1038/s41598-023-31933-w

**Published:** 2023-03-22

**Authors:** Shutong Lai, Olivier Sublemontier, Eric Aubry, Youri Rousseau, Alain Billard, Pascal Briois

**Affiliations:** 1 Institut FEMTO-ST, UMR 6174 CNRS, Université de Franche-Comté, UFC, 2 Place Lucien Tharradin, Site de Montbéliard, 25200 Montbéliard, France; 2 CEA, CNRS, NIMBE, Université Paris Saclay, 91191 Gif-Sur-Yvette Cedex, France; 3 Institut FEMTO-ST, UMR 6174 CNRS, Université de Technologie de Belfort Montbéliard, UTBM, Rue Ernest Thierry Mieg, Site de Montbéliard, 90010 Belfort Cedex, France

**Keywords:** Engineering, Materials science

## Abstract

The incorporation of nanometric-sized objects in conventional coatings can improve the properties of the matrix alone or give rise to new functionalities brought by the nanostructures. Current processes call on various shaping technologies that depend on the nature of the nano-inclusions and the matrix considered. Here, we present an integrated process based on the incorporation of nanoparticles using the aerosol route. It combines divergent nanoparticle jets with a uniform spatial profile and Physical Vapor Deposition (PVD). The chemical nature of the nanoparticles is then independent of that used for the matrix. First samples show that the morphology of nanocomposites is strongly dependent on the particle density in the films. Moreover, using several aerodynamic lens arrays combined with smart masking demonstrate the ability for coating on large surface area (40 cm^2^) substrates. These extended possibilities for developing new types of nanocomposites on any type of substrate and on large surface areas at low temperatures proves to be of strategic interest for various applications.

## Introduction

In recent years, nanocomposite coatings, in the form of nano-objects inserted in a matrix of a different material, are of growing interest because of new properties or new functions brought by the nanostructures^1^. In addition, it is possible to take even better advantage of these effects when they are incorporated in a uniform manner in the matrix. The new opportunities arising from such a development cover many applications to biomedicals, photovoltaics, luxury, photocatalysis, mechanics, magnetism or optics. Furthermore, the incorporation of the nanoobjects in a solid and adhesive matrix limits their probability of dispersion in the environment when used. Their potential risk of dissemination is then greatly limited.

The most commonly used deposition techniques for nanocomposite thin film synthesis, sum up in Table 1, are sol–gel, Physical Vapor Deposition (PVD), Chemical Vapor Deposition (CVD), spray pyrolysis, electrodeposition, and other original ways such as phases separation, hybrid PVD/CVD and transferred arc discharge/PVD processes. While each process exhibits specific advantages and drawbacks (see Table 1), several criteria should be considered for the selection of a deposition technology, such as the applicable conditions, the compatibility of each material, or the synthesis operability^2^. Therefore, the current technological limitation mainly lies in the lack of a simple and controlled way to synthesize nanocomposite films, offering more flexibility in the selection of nanoparticles (nature, size and shape) and the matrix materials. The nanoparticles can be produced by several techniques (precipitation, gas phase, laser pyrolysis, combustion, etc.)^3^. However, their transport from the source to the growing film while preserving their properties (agglomeration, nature (e.g., oxidation), dispersion, etc.) remains a challenge.

**Table 1:** Comparison of different technologies of nanocomposite thin film synthesis.

Synthesis methods	Examples of systems matrix/nanoparticles	Advantages	Inconveniences	References
Sol–gel	SiO_2_/NiO-np; TiO_2_/Au-np; PMMA/SiO_2_-np; PVP/ZnO-np	Low working temperature, high purity and homogeneity, rigorous stoichiometry control, coating on large surface is possible	Long reaction time, limited choice for matrix, necessary to accurately control the process for reproductivity, toxicity of some sols	^4–12^
CVD	TiO_2_/Au-np; Al_2_O_3_/ZrO_2_-np	Good reproducibility, high purity and uniform thickness, good adhesion and high deposition rates	Require high working temperature and reserved for volatile materials, production of potentially toxic byproducts, complex legislation	^4,13–16^
PVD	Si_3_N_4_/Ag-np; TiN/Si_3_N_4_-np	Good reproducibility, high purity and uniform thickness, good adhesion, flexibility for materials	Relatively high cost, low vacuum, high working voltage, difficulty to control the nanoparticle size	^4,17–20^
Spray pyrolysis	ZnS/Cu-np; ZnO/Ag-np	Simple and versatile method, control of morphologies, good productivity on a large scale, deposition under ambient atmosphere	Require high working temperature	^4,21–24^
Electro-deposition	Cu/CNT; Graphene/Au-np	Uniform, good adherent, low cost	Need to control many process parameters, restricted to materials with good electrical conductivity	^25–28^
Phase separation	SiO_2_/Si-np	No direct manipulation of nanoparticles, control of size and quantity of nanoparticles	Annealing in high temperature, need to control the stoichiometry of components, control of size and quantity of nanoparticles	^29,30^
Hybrid processes (PVD/CVD)	TiO_2_/Ag-np; C/Cu-np	A wider range of possible deposition	Contamination of materials, high cost	^31,32^
Nanoparticle jet + PVD	CrN/TiN-np	Independent co-deposition; no direct manipulation of nanoparticles, flexibility of materials, easy control of nanoparticles shape and size	Vacuum technology, price, small surface treated	^33–35^
Our process	Large types of metallic or ceramic matrix/nanoparticles	Same as above, large surface treated possible	Vacuum technology, price	^36,37^

Since the pioneering works of Liu et al.^38^ demonstrated the feasibility to manipulate nanoparticles using only aerodynamic effects induced by nozzles^39^, aerodynamic lenses have been largely implemented for aerosol sampling in mass spectrometry^40^, and more recently in cluster beam deposition in which the nanoparticles have been produced in the gas phase^41,42^. In previous works, nanoparticle jets were successfully combined with magnetron sputtering in a single-step process for nanocomposite film synthesis^33–35,43^. In these studies, a jet of nanoparticles was delivered by an adapted classical aerodynamic lens system. While the possible combination of both technologies was demonstrated, only relatively small surface areas could be covered.

In this article, the relevance of the combination of nanoparticles aerodynamic jet with a PVD technology as a single-step process for nanocomposite film synthesis to treat large surface areas is discussed. The production of a divergent jet of nanoparticles is successfully achieved from the expansion of a particle-carrier gas mixture through a modified acceleration nozzle of a classical aerodynamic lens system. The adjustment of the acceleration nozzle diameter, i.e., the last diaphragm diameter in the aerodynamic lens results in a more divergent nanoparticle jet, increasing dramatically the treated surface area. The aerodynamic lens is then an efficient way to transport the nanoparticles from any kind of source at atmospheric pressure to the deposition chamber at a pressure that is compatible with magnetron sputtering. This leads to the nanocomposite thin film formation constituted by metal ceramic or even polymer nanoparticles embedded in a sputtered metallic, or ceramic film. After a brief description of jet formation through the aerodynamic lens and the combination of a nanoparticle divergent jet with magnetron sputtering, some examples of nanocomposite thin film synthesis are presented. Finally, technological improvements for increasing the treated surface are demonstrated.

## Aerodynamic control of divergent and homogeneous nanoparticle jets

Many studies have already been carried out to produce a collimated or very little divergent jet of nanoparticles in a wide range of sizes and with optimal transmission (~ 100%)^44^. The aerodynamic lens is probably one of the most effective devices, both in terms of the particle density in the jet obtained and that of the transmission. Especially, aerodynamic lenses are already used in industry to characterize nanometric or micrometric aerosols by time-of-flight mass spectrometry^45^. The classical aerodynamic lens consists of several thin plate diaphragms with increasingly smaller aperture diameters which allows the transport of the aerosol from the source to the growing film. The nanoparticle trajectories get closer and closer to the propagation axis along their travel in the lens, and an accurate control of their spatial and mass distribution can then be obtained. We propose here to extend the classical field of application of aerodynamic lenses to the production of homogeneous nanostructured deposits on large surfaces. The idea is to control the divergence of the jet of nanoparticles by adjusting only the diameter of the acceleration of the aerodynamic lens, the rest of the geometry of this lens remaining unchanged. The control of the nanoparticle jet divergence can be performed on a wide variety of nanoparticles (material, size, shape, …) with near 100% transmission in a wide size range. In this way, it is possible to produce jets of divergent nanoparticles, with a controlled angle of divergence, applicable to the elaboration of homogeneous nanostructured deposits on large surfaces. Numerical calculations carried out using the Flow EFD software (Siemens) were compared to experimental observations. These results are developed elsewhere^46^. Briefly, we observed that it is possible to dramatically increase the divergence of the nanoparticle beam by decreasing the diameter of the acceleration nozzle from the classical value that is used for a classical collimated jet aerodynamic lens. Moreover, by carefully adjusting the volume flow rate in the lens with the help of a critical orifice inserted at the lens entrance, homogenous deposition of particles can be achieved.

**Figure 1:**
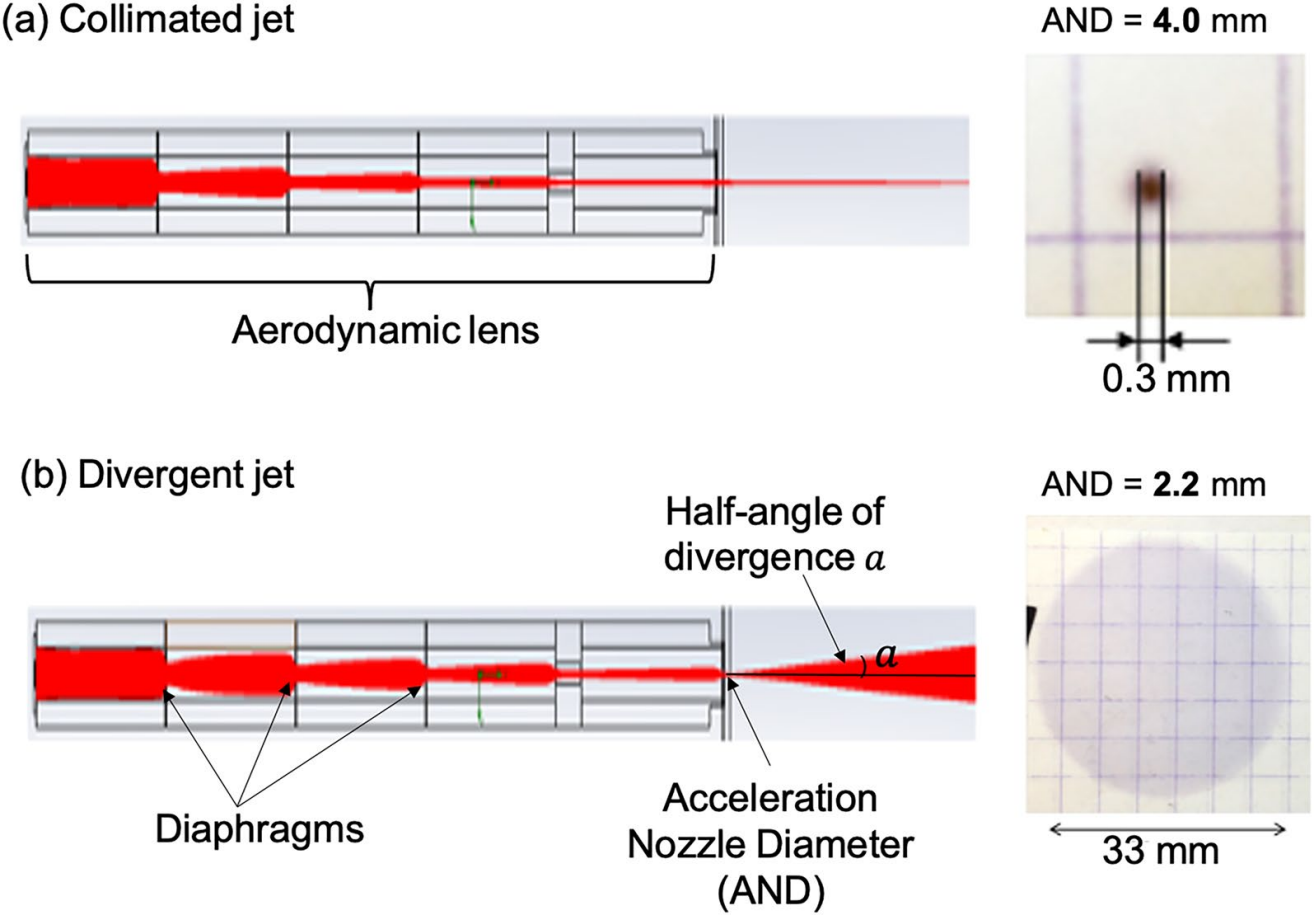
Numerical simulations of trajectories of 15 nm gold nanoparticles (red) in an aerodynamic lens (left side) and corresponding experimental results (right side) of simple nanoparticles deposits on a paper substrate for two different acceleration nozzle diameters (AND) (4 mm in (**a**) and 2.2 mm in (**b**)).

In Fig. 1, an example of the results of numerical calculations in two configurations is given. In Fig. 1a are represented trajectories of nanoparticles (red) under conditions of a collimated jet with 15 nm gold nanoparticles. Figure 1b are shown trajectories of nanoparticles under divergent jet conditions with the same nanoparticles. The corresponding deposits made experimentally with similar real nanoparticles on a paper substrate placed at 260 mm from the outlet of the aerodynamic lens are also presented in each case on the right side. With a diameter of the acceleration nozzle of 4 mm, the observed deposit is very sharp, with only a few tenths of a millimeter in diameter. While using a smaller diameter of 2.2 mm, a 100-time larger diameter of more than 30 mm can be covered with nanoparticles in a homogeneous deposit. Details of the divergence angle as a function of the acceleration nozzle diameter for different types of particles are given elsewhere [O. Sublemontier et al. submission process in progress]^46^.

## Combination of nanoparticles divergent jet with magnetron sputtering

**Figure 2:**
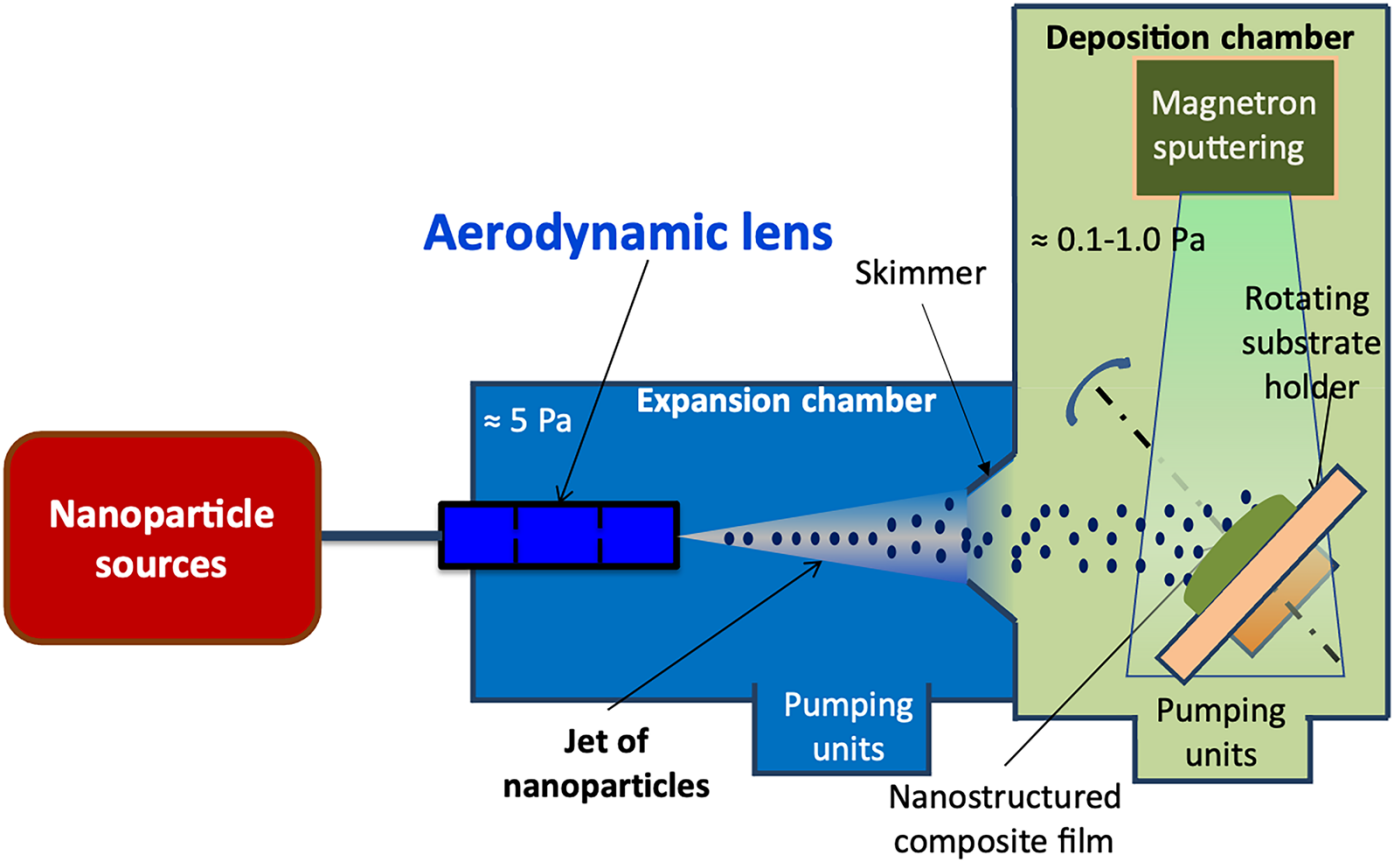
Scheme of the nanocomposite thin film process constituted by a source of nanoparticles connected to an expansion chamber equipped with an aerodynamic lens allowing the transfer of the nanoparticles to the sputtering reactor.

As shown in Fig. 2, the principle of the process lies in the combination of a divergent aerodynamic jet of nanoparticles with a classical magnetron sputtering deposition system. This vacuum process consists of three parts with different pressure levels: the source of nanoparticles at atmospheric pressure, the expansion chamber containing the aerodynamic lens (≈ 5 Pa), and the sputtering chamber equipped with magnetron cathodes and a substrate-holder (≈ 0.1–1 Pa). With the use of adequate differential pumping, composed of a multistage roots primary pump of 40 m^3^/h in the expansion chamber and a 2000 l/s turbo pump in the deposition chamber, the correct vacuum levels are maintained successfully in the two separated chambers.

The application of aerodynamic lenses allows the transport of nanoparticles to be carried out independently. Thus, different nanoparticle sources can be considered as soon as a dry aerosol is formed at the inlet of the aerodynamic lens. It is possible to use either a colloidal suspension that is atomized by a classical aerosol generator (AGK 2000, *PALAS*) to obtain a jet of aerosols under vacuum, or directly from a gas phase nanoparticle synthesis technique such as combustion, inductive plasma, or even laser pyrolysis. Afterward, nanoparticles are aerodynamically driven to the inlet of the aerodynamic lens located in the expansion chamber to achieve nanoparticle delivery. The divergent jet of nanoparticles is formed at the level of the accelerating nozzle at the end of the aerodynamic lens. Furthermore, the propagation through every diaphragm of the aerodynamic lens allows the reduction of the gas pressure at the outlet of the lens to a pressure compatible with that of the sputtering chamber. The role of the expansion chamber is to pump the main part of the gas coming out from the aerodynamic lens.

In a previous work^37^, it was shown that the flow rate of nanoparticles arriving at the substrate-holder decreases strongly with increasing the deposition pressure beyond a value depending on the mass and diameter of the nanoparticle. While the deposition process of the matrix could be carried out with different technology, magnetron sputtering has been privileged for its robustness, its large use in the industry, and other advantages such as a compatible vacuum level and deposition rate, flexibility concerning the choice of possible materials and a matrix deposition at ambient temperature. Besides, the high nanoparticles deposition rate is also compatible with that achieved in magnetron sputtering technologies (from few to tens nm min^−1^)^47^.

It is noteworthy that this combination results in a safe-by-design process because of no direct manipulation of nano-object, in a single-step process with simultaneous deposition of the nanoparticles and the matrix, and a versatile process offered by the independent deposition of the nanoparticles and the matrix.

### Nanocomposite films synthesis

As different choices for the nanoparticle source are possible, depending on the type of the desired nanoparticle (chemical composition, shape, size, sheath, etc.), the nature and the properties of the matrix films are related to the sputtering parameters, i.e., the nature of the target material (alloy or ceramic) or the possible introduction of the reactive gas. Indeed, by adjusting the cathode electrical parameters^48^, the chemistry of the target surface in implementing reactive gas pulses^49,50^ or plasma emission monitoring^51^, rather precise control of the film stoichiometry can be achieved, which greatly expands the possibilities for the synthesis of nanocomposite thin film materials.

**Figure 3:**
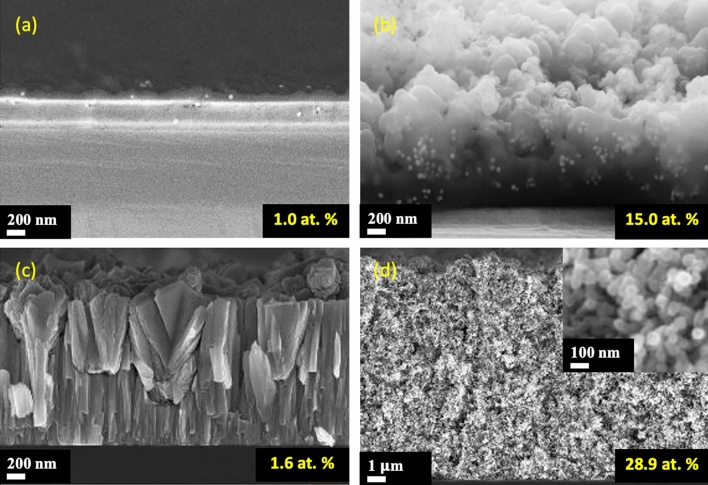
Metallic nanoparticles in a ceramic matrix (Au-np in SiO_2_ matrix) with 1 at.% np (**a**) and 15 at.% np (**b**); ceramic nanoparticles in a metallic matrix (Si-np in Cr matrix) with 1.6 at.% np (**c**) and 28.9 at.% np (**d**).

To highlight this feature, a selection of different nanocomposite films is shown in Fig. 3. For example, the insertion of ceramic or metal nanoparticles in a metal or ceramic matrix is easily synthesized using the combination of divergent jets with reactive magnetron sputtering.

Furthermore, the control of the nanoparticle density in the matrix ([np]/[matrix]) is a crucial point for the properties of the nanocomposite film. This feature is adjustable either by acting on the nanoparticles concentration in the source or by varying the deposition rate of the matrix.

Changing the concentration of nanoparticles in the source will lead directly to that of [np]/[matrix] in the growing film, assuming no change in their agglomeration during their transport, nor their behavior in the aerodynamic lens. The [np]/[matrix] ratio can also be adjusted by varying the acceleration nozzle diameter in the aerodynamic lens, and so the surface area of the deposit on the substrate.

As shown in Fig. 3, the [np]/[matrix] ratio is as low as 1 at. % or as high as 28.9 at.% can be obtained. It is also worth noting that when the value is very low, the introduction of nanoparticles has a small effect on the film growth and on the resulting morphology, as shown in Fig. 3a,c. However, the coating morphology is strongly disturbed at high nanoparticles concentration, as shown in Fig. 3b,d. Basically, each nanoparticle can be considered as an impurity particle (tens to hundreds of nm) acting as a seed and then leading to nodular growth and the formation of growth defects^52,53^. These defects usually form a hillock shape or nodules which the boundaries are loosely connected to the film, contrary to the columnar growth^54^. According to the nanoparticles flux incoming to the growing layer, the probability for a new impurity particle to condense on already existing growth defects can be relatively high, and consequently limited the nodular growth because of the formation of new nodular growth. The initial nodular growth itself is disturbed. A very considerable number of nodules are loosely connected together thus leading to a dramatic change in the morphology and thickness of the film.

**Figure 4:**
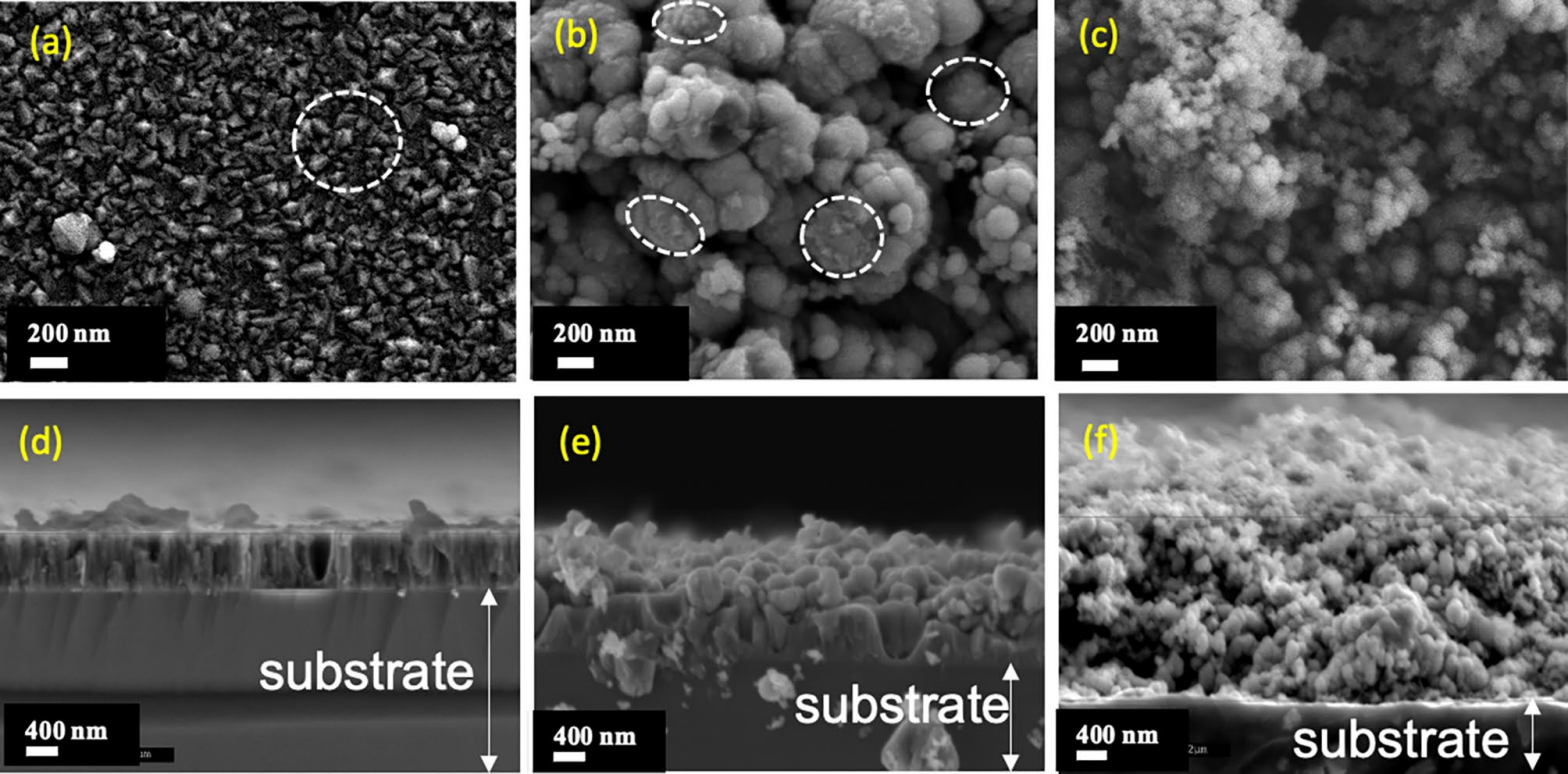
Topographies of TiO_2_/SiO_2_-np films synthesized from different concentrations of SiO_2_-np suspension: (**a**) 0.0 g l^−1^ SiO_2_-np suspension; (**b**) 0.1 g l^−1^ SiO_2_-np suspension; (**c**) 1.0 g l^−1^ SiO_2_-np suspension, and cross-section images: (**d**) 0.0 g l^−1^ SiO_2_-np suspension; (**e**) 0.1 g l^−1^ SiO_2_-np suspension; (**f**) 1.0 g l^−1^ SiO_2_-np suspension.

As introduced previously, it seems reasonable to believe that the addition of a nanoparticle, which can be considered as an impurity, into the growing film would disturb its nucleation and then its growth mode leading to changes in the film morphology and crystallization. Figure 4 shows the evolution of the topography and cross-section images of TiO_2_/SiO_2_-np nanocomposite films synthesized from different concentrations of SiO_2_-np suspensions (0.0, 0.1, and 1.0 g l^−1^) with the same matrix deposition parameters. The size of the SiO_2_-np is about 90 nm. Without the addition of SiO_2_ nanoparticles, the surface of the TiO_2_ film is formed by facets and exhibits a rather moderate roughness (~ 100 nm) corresponding to zone 2 of the well-known structural zone diagram^55^, in agreement with the deposition conditions used here (the substrate temperature reached about 300 °C, ~ 0.5 Pa). The introduction of nanoparticles changes drastically the film topography making it rougher. For the film synthesized with a nanoparticle suspension concentration of 0.1 g l^−1^ SiO_2_, the presence of protrusions having some facets on their tops can be distinguished (circled in white). The lateral size of the protrusion is about a few hundred of nm. The cauliflower morphology is formed with very well-separated protrusions leading to open, porous and, rough film. The estimation of the roughness being impossible with techniques such as Atomic Force Microscopy, it is believed that it would reach more than a few hundred nm. For the film synthesized with a nanoparticle suspension concentration of 1.0 g l^−1^ SiO_2_, this morphological change becomes even more obvious. The presence of a large number of nanoparticles severely disturbs the morphology of classically grown films^55,56^. The protrusions seem smaller but taller, probably resulting in a significant specific surface area, and the presence of facets is no more observable.

**Figure 5:**
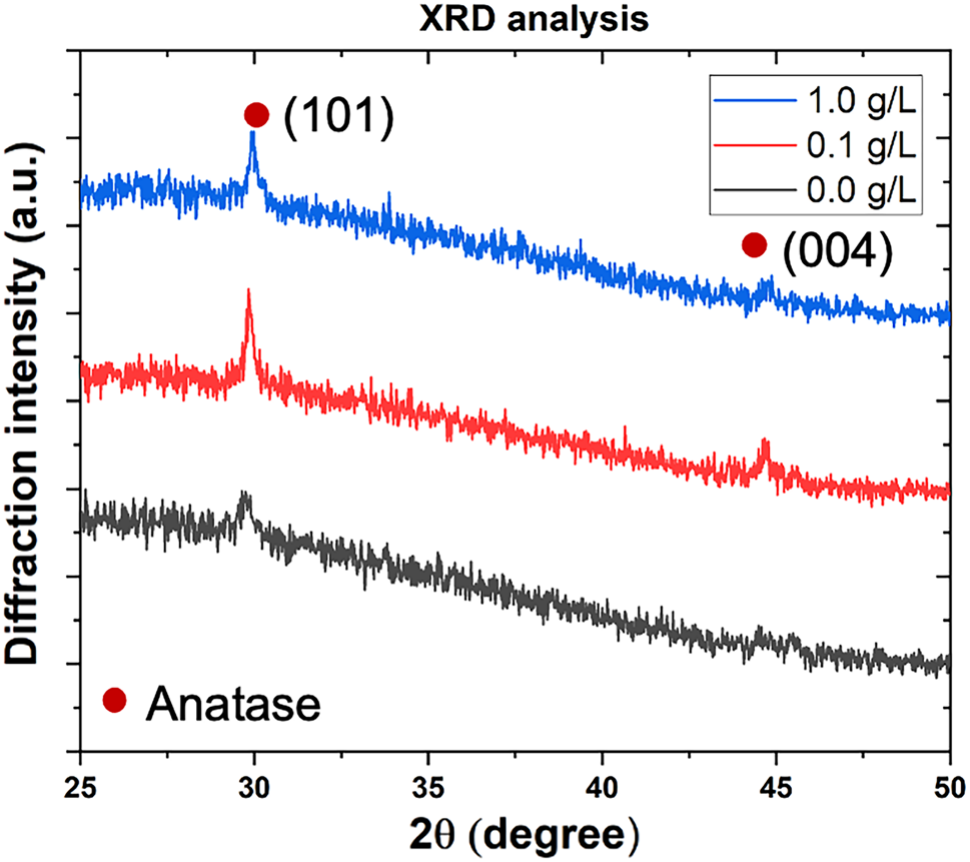
XRD diffractograms of TiO_2_/SiO_2_-np nanocomposite films synthesized from different concentrations of SiO_2_-np suspension.

XRD diffractograms (mode θ–2θ) of the TiO_2_/SiO_2_-np nanocomposite thin films are shown in Fig. 5. Even with a such large difference in morphology, the TiO_2_ matrix structure seems to remain unchanged (same preferential orientation). The X-ray intensity of the diffraction lines does not allow to a deeper investigation of the microstructure. Since SiO_2_-np are amorphous, the peak of SiO_2_-np is not detectable. The incorporation of SiO_2_-np into the growing film disturbs its growth mode leading to an evident change in the film morphology, but the impact on the crystallization of the films seems negligible in this case.

**Figure 6:**
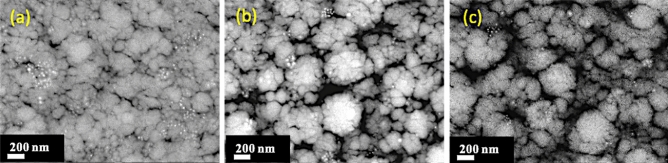
Backscattered electron micrographs of TiO_2_/Au-np nanocomposite films synthesized with increasing the matrix deposition rate via the current intensity applied on Ti targets ((**a**) 2.0 A, (**b**) 2.5 A, and (**c**) 3.0 A.

**Figure 7:**
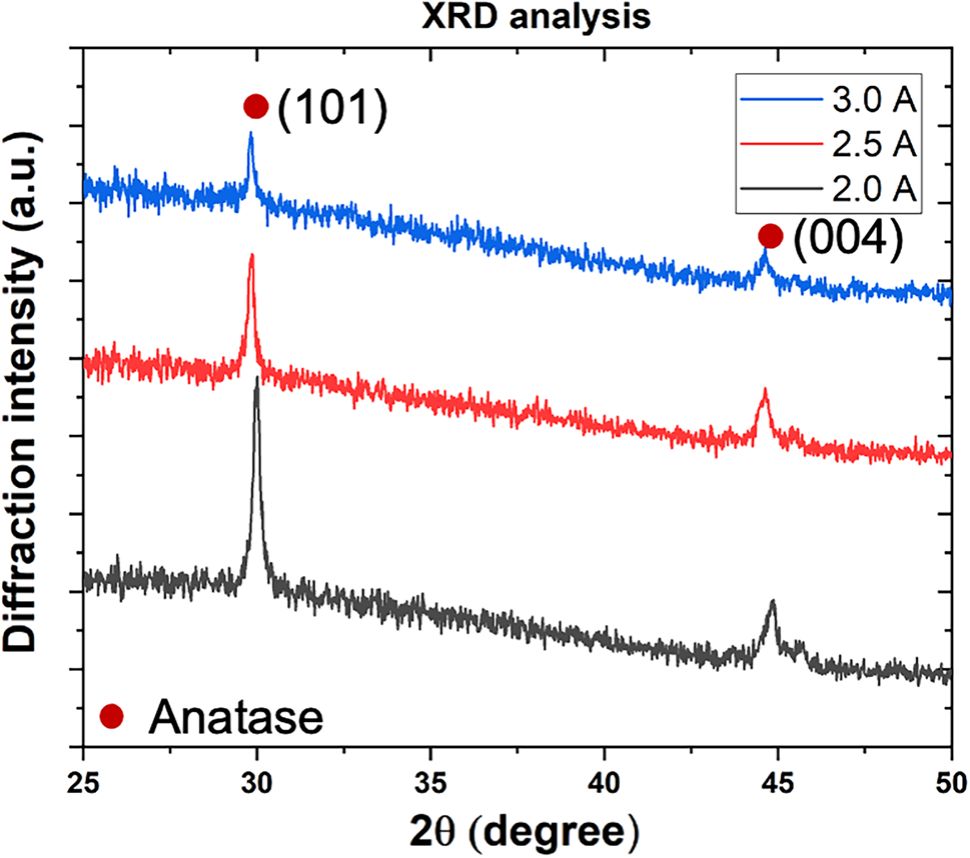
XRD diffractograms of TiO_2_/Au-np nanocomposite films synthesized with increasing the matrix deposition rate via the current intensity applied on Ti targets.

The second way to adjust the [np]/[matrix] ratio is related to the matrix deposition rate. The deposition rate is generally proportional to the electrical power injected in the cathode^57–59^. As an example, Fig. 6 shows the micrographs of TiO_2_/Au-np nanocomposite films sputtered at different current intensities (2.0, 2.5, and 3.0 A). Thanks to the backscattered electron mode revealing atomic number contrast, Au-np could be easily observed (highlighted spots). These 15 nm Au-np were synthesized in our laboratory according to the Turkevich method^60,61^. They were then embedded by a few nanometers shell of SiO_2_ by sol–gel technique right after the synthesis process. This allows the elimination of the surfactant molecules by centrifugation while keeping a stable suspension in ethanol. Au-np are randomly distributed in the deposits it is because the concentration of Au-np suspension is remained low to avoid Au-np agglomerates composed of primary Au-np. They are mainly formed during the drying process of the atomized droplets from the aerosol generator. Figure 7 shows the structural properties (mode θ–2θ) of these samples. The TiO_2_ matrix of the samples is crystallized as anatase in plan (101) and plan (004) and no peak of Au-np is observed for its insufficient content in the layer. For a constant Au-np flow rate, a higher relative TiO_2_ matrix deposition rate leads to a lower relative concentration of gold nanoparticles. Furthermore, for pressures high enough to reduce the mean free path to only a few centimeters (less than the distance from the target to the substrate), the transport of sputtered atoms is driven by gas-phase diffusion^62^. The matrix deposition rate can then strongly depend on the sputtering pressure. A too high sputtering pressure can also affect the transport of nanoparticles to the substrate according to their mass and size. Finally, other ways like hindering the outlet of nanoparticles or sputtering through a grid can only lead to a decrease in their respective deposition rates without changing the deposition parameters (nanoparticles distribution or agglomeration or sputtered species energy).

To sum up, this process enables the synthesis of different types of nanocomposite thin films with an easily tunable density of nanoparticles. However, the perturbation of the film growth mode inherent to the introduction of nanoparticles leads to the formation of surface protrusions and rough and porous films. While the porosity of the films could be first considered as a drawback, applications that require large specific surface area materials, such as photocatalysis, biomedical, or sensors^63,64^, could take a serious advantage from it.

## Towards a process for nanocomposite thin films synthesis on large surfaces

**Figure 8:**
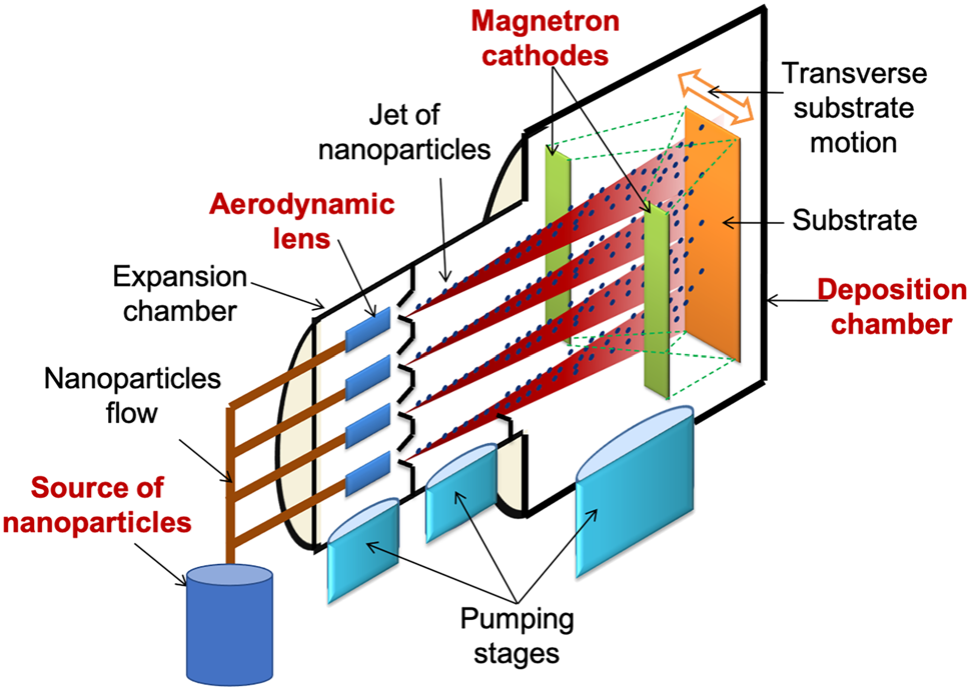
Scheme of a prototype reactor combining a source of nanoparticles, an expansion chamber equipped with four aerodynamic lenses vertically positioned, and a sputtering deposition chamber in which the motion of the substrate-holder is transversal to the nanoparticles.

In order to respond to the needs of industrial applications that require large surface area coatings^65,66^, different setup configurations can be designed based on the principles of the process described previously. For this purpose, a demonstrator, shown in Fig. 8, designed for surfaces of the order of 100 × 100 mm^2^ was designed and produced. The use of several aerodynamic lenses in parallel makes it possible to ensure good coverage of a larger surface area substrate by the overall flow of nanoparticles. Each aerodynamic lens produces a jet of nanoparticles with the same divergence half-angle. The diameter of the acceleration nozzle as well as its distance from the deposition chamber is adjustable according to the half-angle of divergence of the jets, depending on the nature and size of the nanoparticles. Two or even more sputtering cathodes adjustable in distance and orientation are used, and the substrate holder and sputtering sources can move laterally. Furthermore, the use of two cathodes allows to alternate or simultaneously deposit several materials extending the possibility of the matrix choices (e.g., alloys, multilayer, etc.).

**Figure 9:**
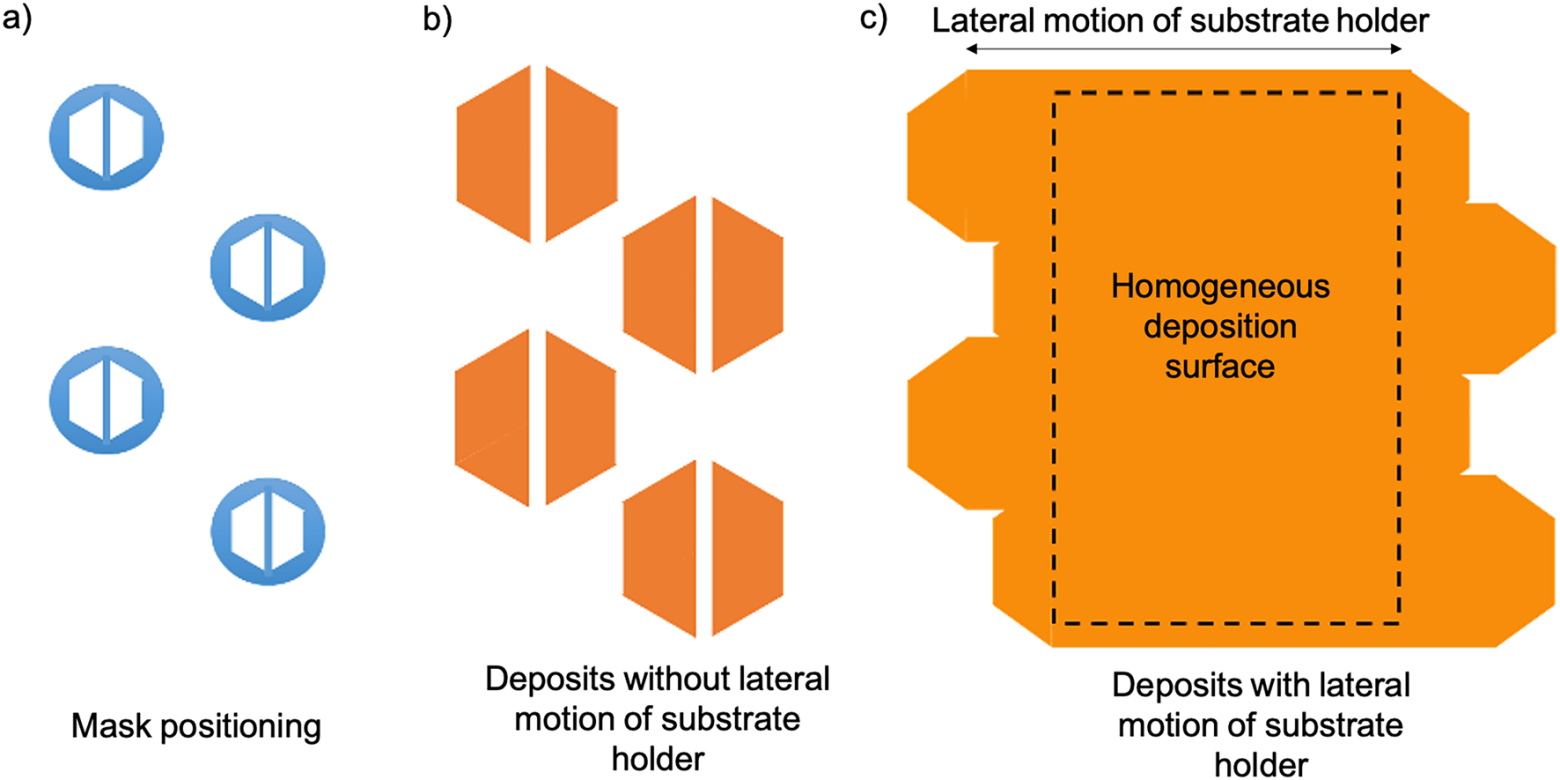
(**a**) Positioning and shape of the masks placed in front of the lens outlets, (**b**) shape of the corresponding deposit of nanoparticles on the substrate without lateral motion of the substrate-holder, and (**c**) shape of the deposit of nanoparticles on the substrate with the lateral motion of the substrate-holder.

The homogeneity of the nanoparticle deposit is obtained by a hexagonal-shape masking of each nanoparticle jet, presented in Fig. 9, so that a homogeneous deposit in thickness and in density of nanoparticles over a large surface area can be achieved with a lateral displacement of the substrate holder. The lenses and the dedicated masks are placed in staggered rows (Fig. 9a) in order to get a hexagonal-shaped deposit for each nanoparticle beam (Fig. 9b). A vertical center line is also added to the masked part in order to eliminate an eventual over-intensity of the nanoparticle jet on its center, due to partial excess of agglomeration of nanoparticles. More details about masking and the nanoparticle jets are given in supplementary information. If the deposits from all the nanoparticle jets are correctly placed on the substrate, the lateral translation of the substrate holder allows a homogenous deposition of the nanoparticles on a surface area (Fig. 9c) that depends on the number of aerodynamic lenses used and the amplitude of the translation. Details about masking and positioning on the nanoparticle jets are given in supporting information.

**Figure 10:**
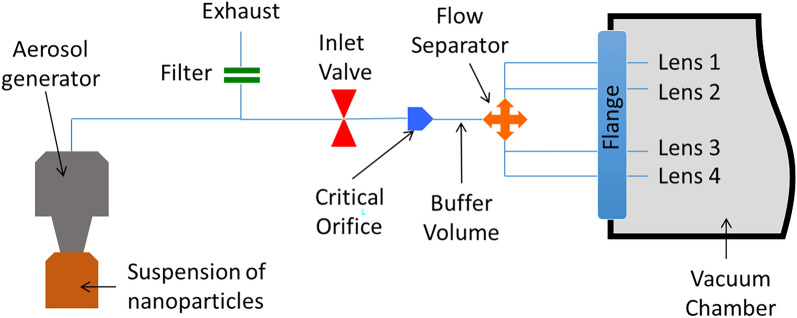
Scheme of the transport of the nanoparticles from the aerosol generator to the four aerodynamic lenses.

The first test of homogeneity on a large surface with four aerodynamic lenses was conducted with a suspension of 30 nm Si nanoparticles previously synthesized by laser pyrolysis. The suspension is loaded with 0.5 g l^−1^ of nanoparticles in Ethanol. After atomization in a standard aerosol generator (AGK 2000, *PALAS*), the flow is separated into four parts by a homemade flow separator based on a simple tube right with angle connections. As shown in Fig. 10, representing the injection of aerosols in the aerodynamic lenses, a single critical orifice of 300 µm diameter was placed before the flow separator. The flow rate with argon as the carrier gas in each aerodynamic lens was regulated at about 0.25 Pa m^3^ s^−1^. An exhaust equipped with a filter is used to throw out the excess flow from the aerosol generator. A buffer volume (60 cm long and 25 mm in diameter) is necessary after the critical orifice to stabilize the aerosol flow before separation. Each aerodynamic lens is a classical aerodynamic lens from Liu^38^ with a modified acceleration nozzle to get a divergent beam of particles with a homogenous spatial profile. In the case of 30 nm Si nanoparticles, an acceleration nozzle of 2.4 mm is required (instead of 4 mm for a classical focusing aerodynamic lens) to get a 4° half-angle divergence. Note that the homogeneity of the deposit depends on the combination of the acceleration nozzle diameter with the critical orifice diameter which determines the volume flow rate at the inlet of the aerodynamic lens (Ar+ nanoparticles). This combination should be slightly adjusted for a given type of nanoparticles (size and density of the material).

**Figure 11:**
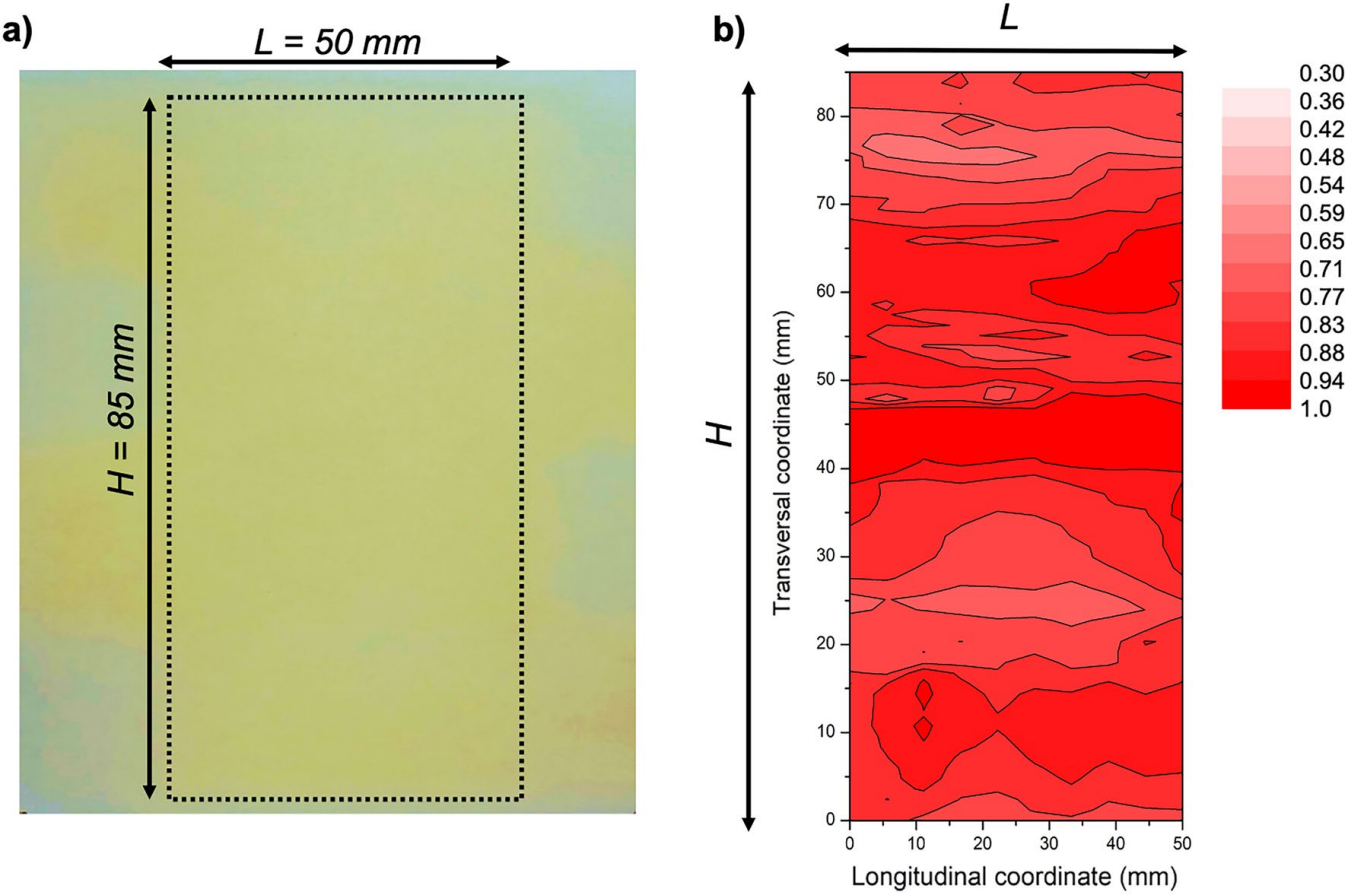
Example of nanoparticles deposit on the large surface area using four aerodynamic lenses, with 30 nm Si nanoparticles. (**a**) Deposition on a paper substrate with the lateral motion of the substrate holder. (**b**) Relative thickness map of the deposit realized in the same conditions on a transparent substrate (color scale corresponds to the relative thickness). The relative thickness variation is below 8% over the whole surface.

Prior to the deposition of the nanoparticles using the four aerodynamic lenses, the homogeneity of the deposit formed by the nanoparticles alone was checked for each aerodynamic lens. The deposit using the four aerodynamic lenses in parallel is presented in Fig. 11. The time deposition was fixed at 75 min. The substrate-holder was moved laterally in a back-and-forth movement of 12 cm amplitude with a constant velocity and a period of 52 s. The deposit presented in Fig. 11a was realized on a paper substrate. Its general shape is consistent with what was expected (see Fig. 9c). A clear homogenous zone appears with a height of 85 mm and a width of 50 mm, confirming the rather good distribution of the nanoparticles over the treated surface. Figure 11b represents a laser extinction analysis of the relative deposit thickness. The laser used is a commercial CW diode emitting 5 mW at 532 nm. The film was analyzed by measuring the laser extinction on a 2D matrix of points separated by 1 mm in distance. As the light intensity loss through the film is less than 15%, the film is considered optically thin, and the film thickness variation can then be approached. With this example of configuration, an approximative 40 cm^2^ homogeneous surface area is treated with a thickness standard deviation of less than 8%.

## Conclusions

In this study, an original single-step and versatile process for the synthesis of nanocomposite thin films based on the combination of a divergent jet of nanoparticles with magnetron sputtering technology has been studied for large surface area treatment. A divergent jet of nanoparticles with a homogenous spatial profile can be formed by adjusting the diameter of the acceleration nozzle of a classical aerodynamic lens, leading to a homogenous deposition of nanoparticles over a surface area of the order of a few cm^2^. The versatility of the process is demonstrated by embedding metal nanoparticles into a ceramic matrix and vice versa. The concentration of the nanoparticles in the coatings is easily varied by adjusting the concentration in the nanoparticles sources, or by changing the matrix deposition rate. Due to the simultaneous deposition of nanoparticles and matrix, the morphology of the thin films and consequently their porosity and roughness are closely related to the density of nanoparticles. Furthermore, the combination of a multi-aerodynamic-lens system with the smart masking of the corresponding jets and the translation of the substrate holder offers large surface areas processing, demonstrating the compatibility of the process with demanding industry requirements. Future work will focus on the optimization of the process to have a better homogeneity of the films by controlling particle agglomeration, and more precise structural analysis of the nanocomposite materials. The exploration of new applications is already underway with new nanoparticle/matrix couples while there is no limitation in the choice of their respective compositions.

## Supplementary Information

Supplementary Information.

## Data Availability

The datasets used and/or analyzed during the current study available from the corresponding author on reasonable request.
