# Supplementary material for: Aerosol-based functional nanocomposite coating process for large surface areas
Source: Sci Rep. 2023 Mar 22;13:4709. doi: 10.1038/s41598-023-31933-w (PMC10033632; doi:10.1038/s41598-023-31933-w)
Supplement: Supplementary file 1 — Supplementary Information. [file 41598_2023_31933_MOESM1_ESM.docx]

Supporting information

Fig. S1 and Fig. S2 schematically show the layout of the various technical elements. The first figure is a top view representation. The second is a side view representation. A set of 4 aerodynamic lenses is shown as an example. The number of aerodynamic lenses to use depends on the surface area to be covered. Each of these aerodynamic lenses produces a jet of nanoparticles, which main characteristic is its half-angle of divergence α. The jets are produced in an expansion chamber equipped with pumping systems (primary pump + multi-stage roots, for example) making it possible to reach a pressure of the order of or less than 1 mbar. They then pass through a skimmer to isolate the expansion chamber from the deposition chamber from the vacuum point of view while allowing all the particle jets to pass. The diameter of the skimmers as well as their distance from the exit of the aerodynamic lenses is adjustable according to the half-angle of divergence of the jets of particles. The deposition chamber is maintained under vacuum by appropriate pumping means, for example a turbomolecular pump, up to pressures compatible with the means used for deposition of the matrix. For example, in the case of the use of conventional magnetron sputtering, the pressure will be maintained at a value of the order of 5.10^-3^ mbars. A pressure lower than 5.10^-2^ mbars is desirable to guarantee the integrity of the path of the particles through the deposition chamber to the substrate over a distance of several tens of cm (see previous patent). At the entrance to the deposition chamber, each of the particle jets passes through a mask whose geometry is identical for all the jets and whose role is to spatially filter part of the particles so as to impart a well-defined geometry to the deposit. Sources of material for the matrix (for example magnetron cathodes) are placed symmetrically on either side of the nanoparticle jets. In this example, there are 2 of them but they can be more numerous. These sources of material make it possible to cover, at the same time as the nanoparticles, part of the substrate in top view and the entire surface to be covered in side view. They can therefore have an oblong shape as shown in the diagram. The substrate is mounted on a substrate holder which can be heated and/or polarized and which is driven, thanks to motorized equipment, in a horizontal translation movement, perpendicular to the central axis of the particle jets.


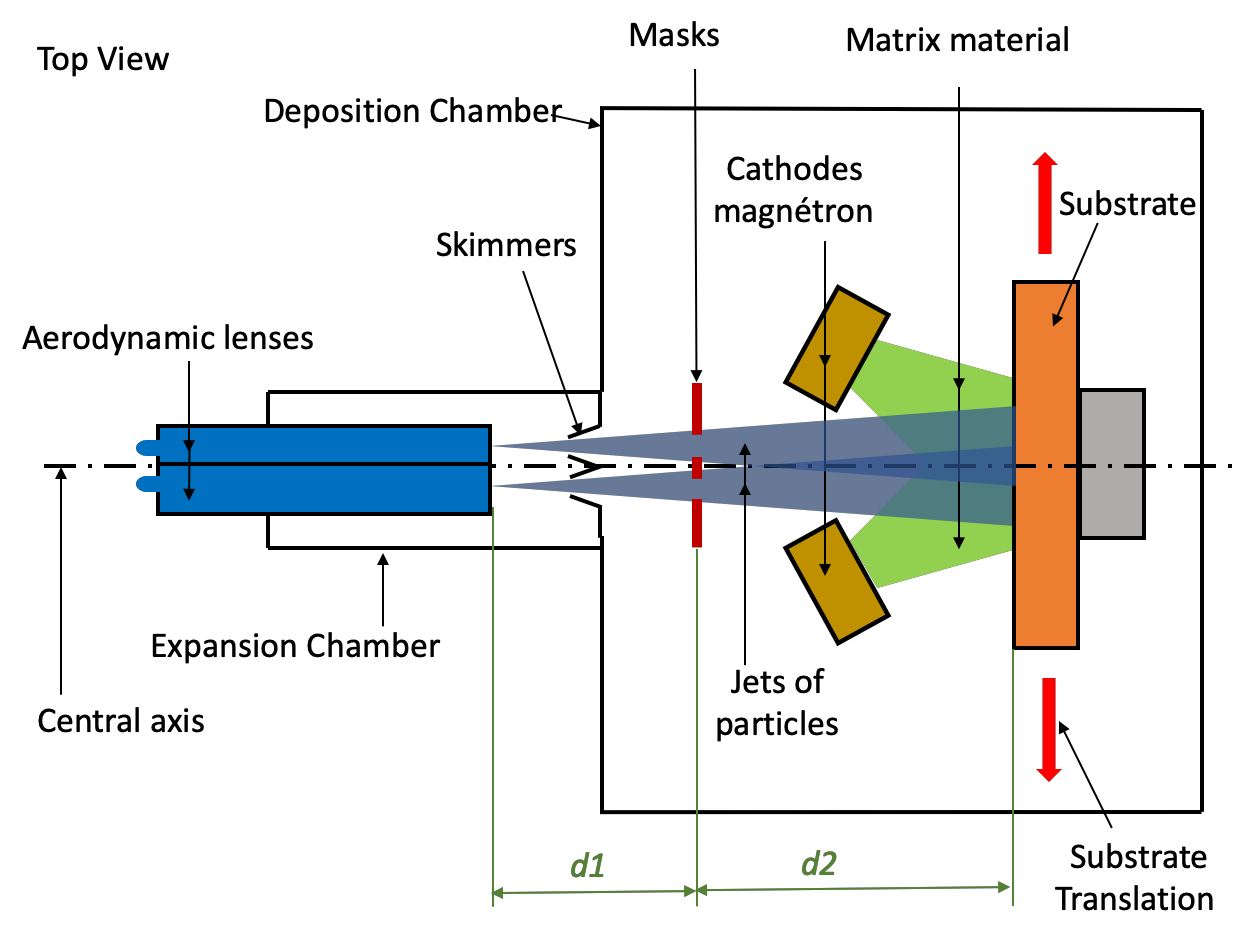


*Figure S1: Layout of the different technical elements in top view*


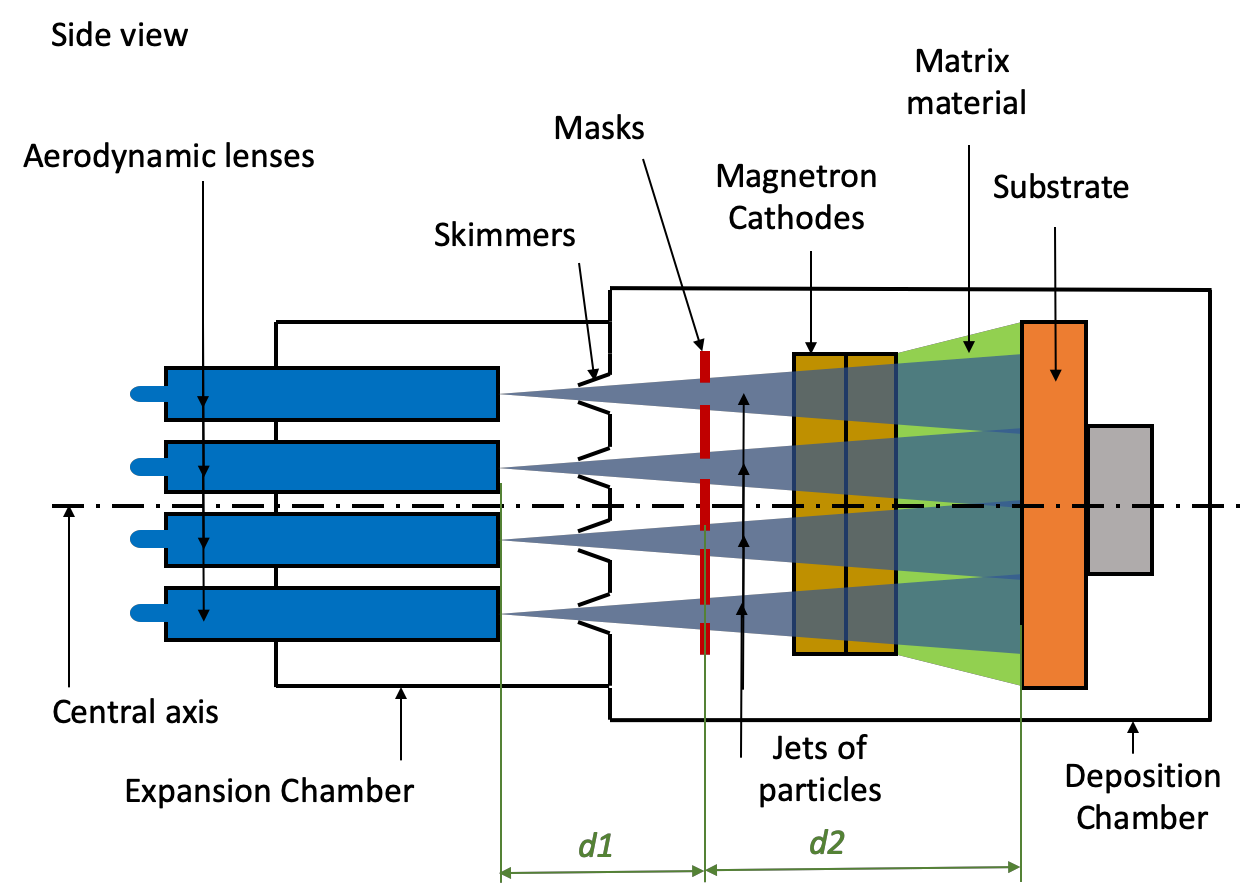


*Figure S2: Layout of the different technical elements in side view*

The technical solution proposed here consists in using several divergent aerodynamic lenses in parallel. The spatial arrangement of the aerodynamic lenses must be adjusted so as to obtain a homogeneous NP deposition over a large surface. Masking of the particle jets is carried out so as to obtain a homogeneous deposition by lateral translation of the substrate in the plane perpendicular to the main axis of the aerodynamic lenses. This deposit is homogeneous when the deposits from each lens are perfectly juxtaposed on the substrate, or when the projection on the vertical axis of the quantity of deposited particles is constant.

Besides the beam divergence, the homogeneity of the nanoparticle deposition is of prime importance. To estimate this parameter, coatings are deposited on glass substrates. The estimation of the relative film thickness is performed by laser extinction experiments using a laser diode (532 nm) in optically thin conditions (maximum extinction < 15%). Tow aerodynamic parameters are important to get a homogenous deposition. The first one is the acceleration nozzle diameter and the second one is the diameter of the critical orifice, which is placed at the entrance of the aerodynamic lens and define the gas volume flow rate in the lens. Fig. S3 shows an example of relative thickness profile obtained with the deposit of 30nm Si-np, and with optimized aerodynamic parameters (expansion nozzle diameter of 2.2 mm and critical orifice diameter of 220 µm). The homogeneity of the relative thickness in these conditions by our process is confirmed (standard variation < 6 %).


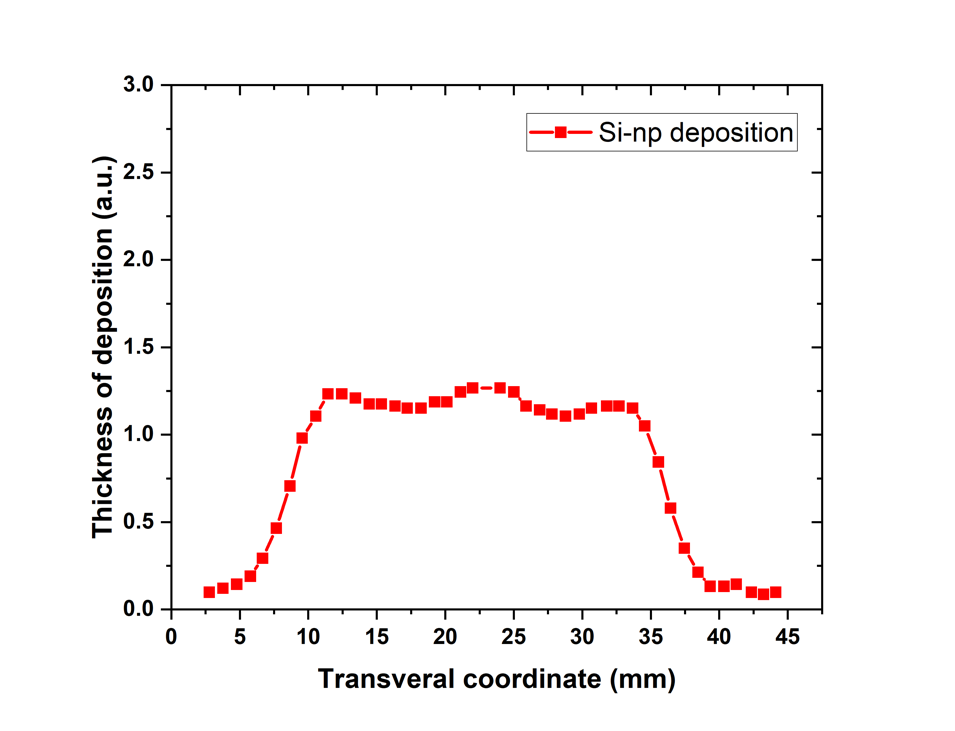

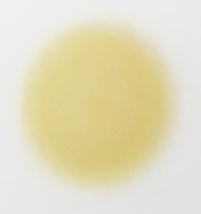


Transversal coordinate

*Fig. S3 Relative thickness of a 30 nm Si-np film with optimized aerodynamic parameters as a function of the transversal coordinate. A photo of the corresponding film is presented on the right.*

Fig. S4 schematically represents the deposits obtained on the plane of the substrate with hexagonal masking. This masking makes it possible to avoid zones of overintensities or under intensities at the junctions between adjacent deposits by having masking allowing a deposit on a larger surface for each lens, and therefore a faster overall particle deposition rate. important. Fig. S4a) is for the detail of a lens. Fig. S4b) is an example including 4 lenses with a staggered arrangement in front view, with 2 lens groups, each aligned on a vertical axis.

*
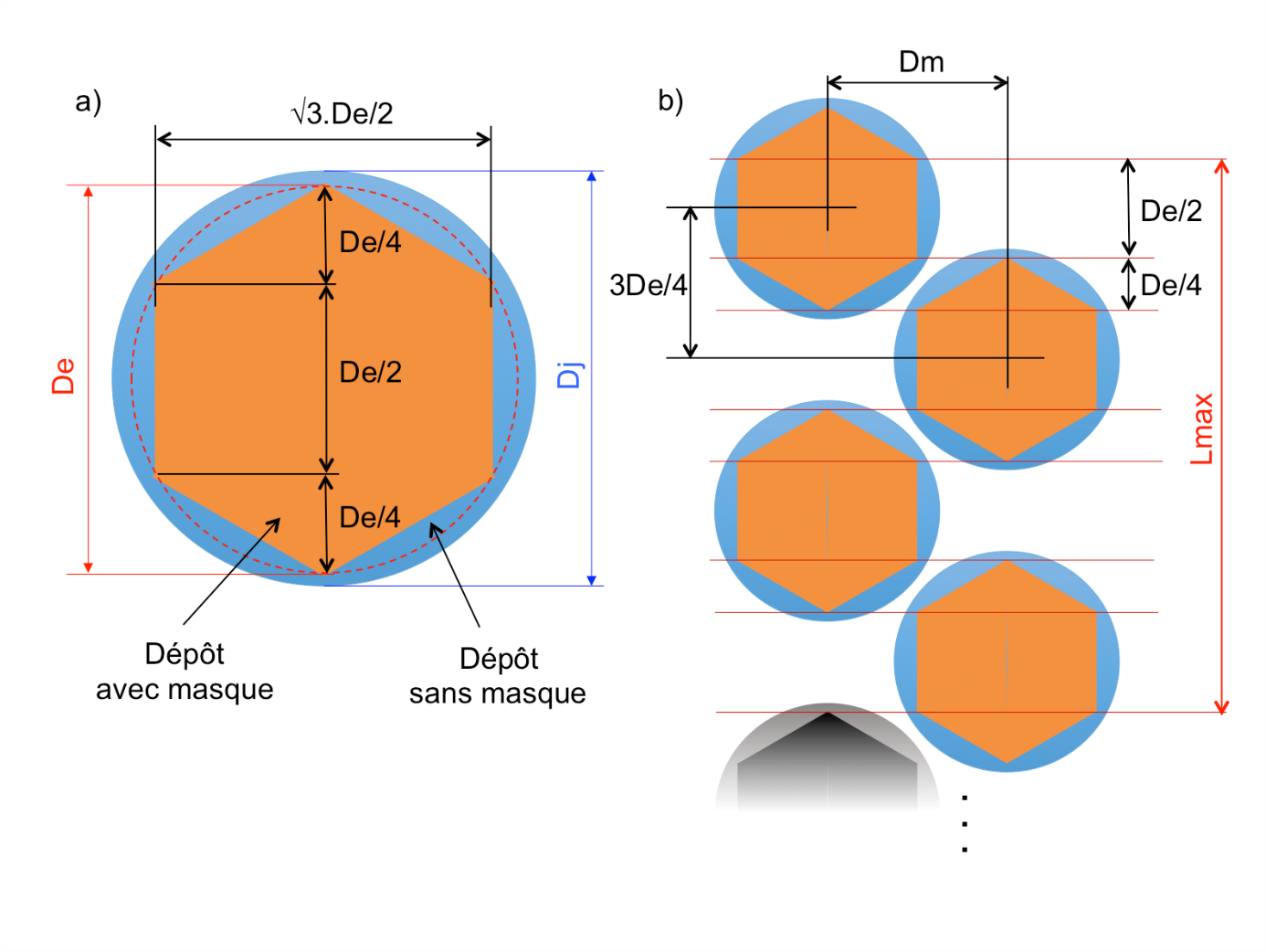
*

*Figure S4: hexagonal masking representation (Front view)*

The central axes of the aerodynamic lenses are all horizontal. This arrangement, associated with the action of the masks, allows homogeneous deposition by horizontal translation of the substrate. To obtain this homogeneous deposit by translation, the masks have a regular and symmetrical hexagonal shape included in a circle of radius De. Only the central part of the jet is allowed to pass (in orange on the figure) and filter the edges (in blue on the figure). To avoid the effects of inhomogeneity in the vicinity of the edges in the masking step, one can for example choose De=0.9.Dj, Dj being the maximum diameter of the deposit. The relationship between Dj and De is to be assessed according to the quality of the edge of the deposit without mask. The hexagons are placed in such a way that they have 2 vertices placed on the vertical axis. We align the low top of the first aerodynamic lens with the high points of the vertical segments of the 2nd aerodynamic lens on the horizontal. We then align the low points of the vertical segments of the 2nd aerodynamic lens with the high top of the 3rd one, and so on. The distance between the horizontal axes of 2 successive aerodynamic lenses is therefore 3.De/4. It follows that the vertical dimension Lmax covered homogeneously by a set of n aerodynamic lenses arranged in this way is: Lmax = (3n-1).De/4 Using 4 aerodynamic lenses, with De=36.8 mm, as in the previous realistic example, the vertical dimension covered evenly is 101.2 mm. The horizontal dimension covered depends only on the displacement capacities of the substrate. It is also possible, in the case of a flexible substrate wound on itself, to cover very large horizontal dimensions. On a rigid substrate, the substrate holder will have to move laterally by a distance equal to the horizontal dimension to be covered + 2(De+Dm), with Dm being the distance between the vertical axes of the 2 aerodynamic lens groups. This distance must be sufficient to avoid covering the deposits of 2 adjacent aerodynamic lenses. By choosing Dm=De, for example, the distance between the axes of 2 adjacent lenses is slightly greater than Dj. No recovery of the deposits will therefore take place in this case, even if the geometry of the masks is modified. In the case of the use of hexagonal masks, this displacement can be reduced by choosing Dm less than De. In this case, it is not necessary to avoid the overlap between the blue zones (in Fig. S4b), but between orange areas of 2 adjacent aerodynamic lenses. Regular hexagonal type masking has several advantages. It allows for an identical pattern across the lens groups, relatively easy to achieve, and allows for minimal particle filtering, allowing for a higher overall particle deposition rate than with diamond or square type masking.

The geometry of the masks is a homothety of ratio f with respect to the geometry of the desired deposits presented in Fig. S4. The ratio f or magnification factor is the ratio d1/(d1+d2), where d1 is the distance between the aerodynamic lens exit and the masks, and d2 is the distance between the mask and the substrate. Note that the distances between the axis of the different LAs are the same as on the deposits in the plane of the substrate in Fig. S4b, these axes being horizontal.
